# Comparison of Thermal Deformation Behavior and Characteristics of Mg-Gd-Y-Zn Alloys with and without Bulk LPSO Phase

**DOI:** 10.3390/ma16175943

**Published:** 2023-08-30

**Authors:** Dongjie Chen, Qi Wang, Liang Zhang, Ting Li, Jiawei Yuan, Guoliang Shi, Xinyu Wang, Kui Zhang, Yongjun Li

**Affiliations:** 1 School of Architecture and Civil Engineering, Huanghuai University, Zhumadian 463000, China; 2 Guobiao (Beijing) Testing & Certification Co., Ltd., Beijing 100088, China; 3 China United Test & Certification Co., Ltd., Beijing 100088, China; 4 State Key Laboratory of Nonferrous Metals and Process, GRINM Group Co., Ltd., Beijing 100088, China; 5 GRIMAT Engineering Institute Co., Ltd., Beijing 101407, China

**Keywords:** Mg-Gd-Y-Zn alloy, thermal deformation behavior, LPSO phase, dynamic recrystallization

## Abstract

Alloys Mg-8Gd-4Y-0.6Zn-0.5Zr (referred to as 0.6Zn) without the bulk long-period stacking ordered (LPSO) phase and Mg-8Gd-4Y-1.1Zn-0.5Zr (referred to as 1.1Zn) containing the bulk LPSO phase were prepared and a series of hot compression tests were conducted to examine and evaluate the influence of the bulk LPSO phase on the thermal deformation behavior and characteristics of the Mg-Gd-Y-Zn-Zr alloy. The bulk LPSO phase affects the dynamic recrystallization behavior, resulting in differences in flow stress between two alloys under different conditions. Specifically, in the temperature range of 380~460 °C, compression at lower strain rates is beneficial for the LPSO phase to promote dynamic recrystallization, while compression at a high strain rate inhibits the dynamic recrystallization due to the severe deformation of the bulk LPSO phase to release the stress concentration instead. The increase in temperature helps the LPSO promote dynamic recrystallization. As a result, the LPSO phase promotes dynamic recrystallization at all experimental strain rates at 500 °C. Furthermore, the thermal processing maps of the 0.6Zn and 1.1Zn alloys are established, and their optimal processing windows are located at 500 °C/0.001~0.01 s^−1^ and 500 °C/0.01 s^−1^, respectively. In addition, the instability zones for the 1.1Zn alloy are much larger than that for the 0.6Zn alloy, which corresponds to the microcracks generated at the interfaces between α-Mg and bulk LPSO phases.

## 1. Introduction

Magnesium alloy is regarded as the most promising structural metal material of the 21st century due to its benefits of abundant reserves, low density, high specific strength and stiffness, and simple recycling, and has been promptly gaining attention in a variety of fields, including aerospace, automotive, and weapons [1,2,3]. However, magnesium alloys usually feature restricted slip systems activated at room temperature because of the close-packed hexagonal structure, which results in low room temperature deformation capacity, making for a generally lower absolute strength and plasticity compared with aluminum and steel alloys [4,5].

Optimizing alloy composition is one substantial way to increase the properties of magnesium alloys [6,7]. Among several magnesium alloy systems that have been extensively studied recently [8,9,10,11,12,13,14,15], the Mg-RE alloy has gained a lot of attention because of its strong aging response and excellent yield strength. Furthermore, by replacing rare earth elements with appropriate amounts of Zn, the raw material costs are decreased, and the resulting alloys exhibit significantly improved mechanical properties [16,17,18]. For example, Xu [16] effectively created a high-strength and high-ductility magnesium alloy by forcing air cooling on the hot extruded alloy at the extrusion outlet and aging treatment. Tensile yield strength is 466 MPa, tensile strength is 514 MPa, and fracture elongation is 14.5%. Actually, Mg-RE-Zn alloy is currently the alloy with the highest strength and superior plasticity obtained through traditional heat treatment and processing, reaching the level of the commercial 7075 aluminum alloy after T6 treatment.

Wrought Mg-RE-Zn alloy, usually conducted at a specific heating temperature for a better deformation, has more advantageous overall characteristics than cast Mg-RE-Zn alloy and may be employed as a load-bearing component to satisfy a wider variety of industrial demands [19,20]. A series of phenomena arises during the thermal deformation, including work hardening, dynamic recovery and recrystallization, which ultimately has an impact on the microstructures and is intimately related to the deformation parameters and the initial microstructure. There are currently several investigators into the thermally deformed Mg-RE-Zn alloys, mainly focusing on the thermal deformation behavior, the microstructure evolution and the mechanism. Wang [21] investigated the thermal deformation behavior of Mg-Gd-Zn alloy and discovered that the flow stress dropped with increasing temperature and strain rate, but the percentage of dynamic recrystallization rose with increasing deformation temperature. Zhang [22] studied the impact of lamellar and bulk long-period stacking ordered (LPSO) phases on the behavior of dynamic recrystallization (DRX), and they discovered that the lamellar LPSO phase suppressed DRX while the bulk one increased DRX. Zheng [23] proposed that the kinking of lamellar and bulk LPSO phases can delay the continuous dynamic recrystallization (CDRX) behavior, but they also suggested that this kinking may activate DRX more strongly through the particle stimulated nucleation (PSN) mechanism. Meanwhile, Li’s study [24] showed that the bulk LPSO phase facilitated continuous dynamic recrystallization due to the decreased inhibition on the movement of dislocations, and the lamellar LPSO phase contributed to discontinuous dynamic recrystallization via the PSN mechanism. Furthermore, Zhou [25] discovered that the activation energy of thermal compression cast Mg-4.9Gd-3.2Y-1.1Zn-0.5Zr (wt.%) alloy was 285 kJ/mol, which was much higher than that of standard Mg-Al, Mg-Zn, and Mg-Gd-Y alloys. According to Tahreen [26], the LPSO phase causes increased activation energy in Mn-Y-Zn-Mn alloys. In addition, Xu’s research [27], on the other hand, reveals that the solid solution of RE elements in the matrix can greatly increase the rheological activation energy of Mg-RE alloys.

Overall, there are still discrepancies in opinions on the thermal compression deformation behavior of Mg-Gd-Y-Zn alloys, particularly regarding the impact of the LPSO phase on activation energy and dynamic recrystallization. In order to clarify the impact of the LPSO phase on alloy thermal deformation, it is necessary to conduct more direct comparative experiments, such as by controlling its content or even its presence to compare and study the role of the LPSO phase. This work aims to explore the effect of bulk LPSO phases on the thermal deformation behavior. With this purpose, two alloys, Mg-8Gd-4Y-0.6Zn-0.5Zr free bulk LPSO phase and Mg-8Gd-4Y-1.1Zn-0.5Zr containing the bulk LPSO phase, were prepared, and thermal compression experiments were carried out to investigate the impact of the bulk LPSO phase on thermal compression deformation characteristics, microstructure evolution as well as thermal workability.

## 2. Materials and Experiments

Cast Mg-8Gd-4Y-0.6Zn-0.5Zr (wt.%) alloy and Mg-8Gd-4Y-1.1Zn-0.5Zr (wt.%) alloy (referred to as 0.6Zn alloy and 1.1Zn alloy in the later text, respectively) with a 400 mm diameter and 800 mm length were made using semi-continuous casting. Ingots were homogenized at 773 K for 48 h and then subjected to water quenching. The phase percentages of two alloys at various temperatures and the heat treatment regime were determined by JmatPro (JmatPro 7.0) software. Thermal compression experiments were conducted on Gleeble-3500 simulation equipment with a strain rate and temperature ranges of 0.001 to 1 s^−1^ and 380 to 500 °C, respectively. After being heated for 3 min at the experimental temperature, the samples were compressed with a 60% height decrease.

**Figure 1:**
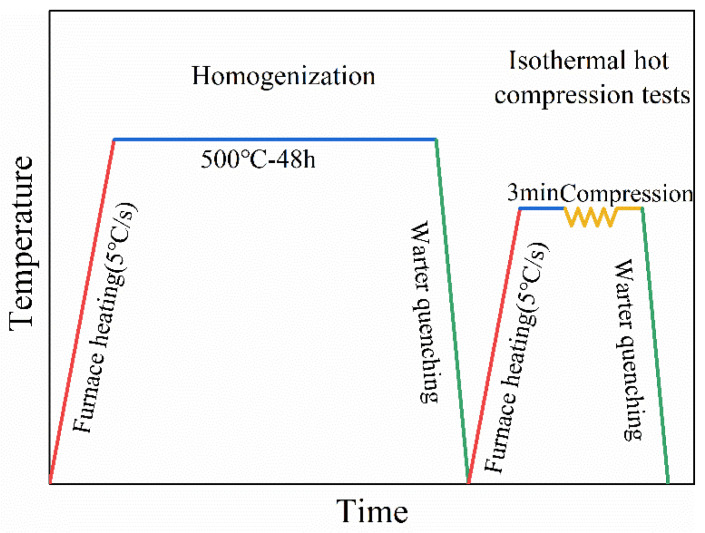
Schematic diagram of solid solution treatment and hot compression process for 0.6Zn and 1.1Zn alloys.

In addition, the specimens were promptly quenched in water after being deformed in order to retain the distorted microstructures. The experimental procedure is depicted in Figure 1.

The microstructure of the compressed specimens on the section’s center was examined after being cut along a centerline parallel to the direction of compression. Metallographic and scanning electron microscope microstructures were obtained using a ZEISS AXIOVERT 2000MAT OM (ZEISS, Tokyo, Japan) and JSM-7900 F SEM (JEOL, Tokyo, Japan), respectively. Phase identification was carried out using X-ray diffraction (XRD) with a Cu target. The sample was scanned through 2θ from 20 to 100° with a scanning rate of 2°/min. X-ray diffraction with a Cu target was used to perform phase identification. The specimen was tested at a rate of 2°/min with 2θ from 10 to 90°.

## 3. Results and Discussion

### 3.1. Initial Microstructure

**Figure 2:**
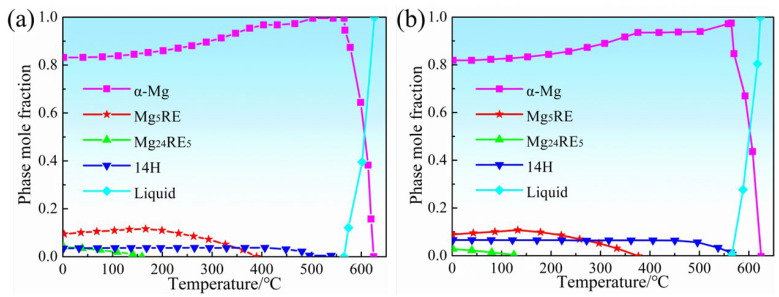
The variation in the mole fraction of various equilibrium phases in the (**a**) 0.6Zn alloy and (**b**) 1.1Zn alloy with increasing temperature, which were calculated by using JMatPro simulation software.

Relationships between the equilibrium phase mole fraction and homogenization temperature in 0.6Zn and 1.1Zn alloys are depicted in Figure 2, as calculated using the JMatPro simulation software. According to the calculated results, the LPSO phases in 0.6Zn alloy are completely dissolved after homogenization heat treatment at 500 °C, whereas there is still about 6.0% of LPSO phases in the 1.1Zn alloy.

**Figure 3:**
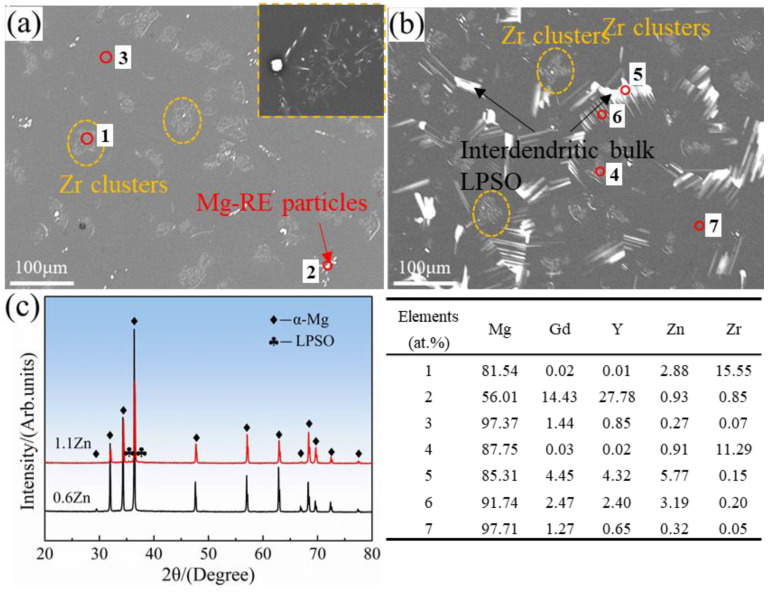
SEM images of homogenized 0.6Zn alloy (**a**) and1.1Zn alloy (**b**), as well as their XRD pattern (**c**). The insert in (**a**) shows the enlarged SEM micrographs of Zr clusters. The element atomic percentages obtained from EDS analysis at different locations are given in the inserted table.

The microstructure and phase composition of homogenized 0.6Zn and 1.1Zn alloys are depicted in Figure 3. There is no LPSO phase in the homogenized 0.6Zn alloy, and only Zr clusters and Mg-RE particles are within the grain and at the grain boundaries, respectively. By contrast, except for Zr clusters with multiple strips in different orientations in the α-Mg matrix (as inserted in Figure 3a), a certain number of bulk LPSO phases are identified in the homogenized 1.1Zn alloy. XRD is a good method for analyzing the phase composition, distribution, and crystallographic structure [28]. According to the XRD patterns in Figure 3c, the big difference between two alloys is the appearance of bulk LPSO phases with the 14H LPSO structure in the 1.1Zn alloy, in agreement with the microstructure observation. However, due to the small amount of the LPSO phase, the LPSO phase content calculated based on XRD results is less than 1%, which is significantly lower than the 4.5% result calculated based on scanning electron microscopy photos. Furthermore, the element atomic percentages obtained from EDS analysis at different locations are given in the inserted table.

### 3.2. Thermal Compression Deformation Behavior

#### 3.2.1. Flow Stress Curves

**Figure 4:**
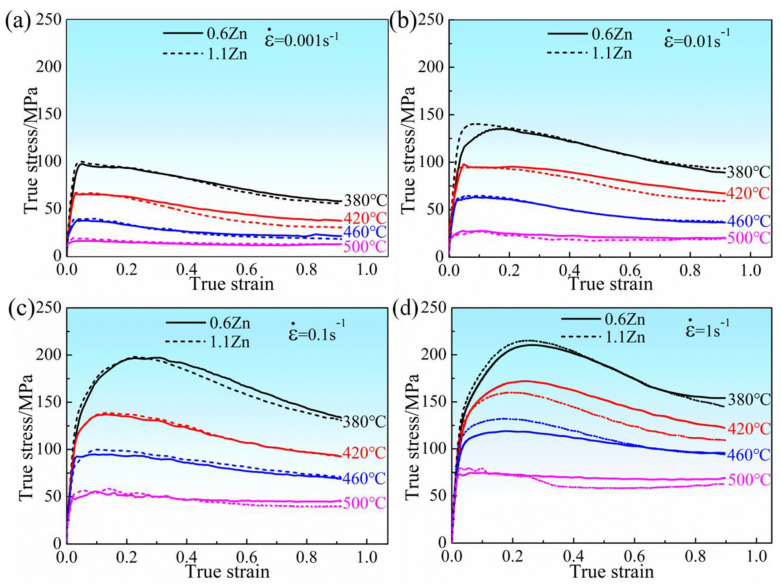
True stress-strain curves for 0.6Zn and 1.1Zn alloys at 380~500 °C under different strain rates (**a**) $\dot{\varepsilon }$ = 0.001 s^−1^; (**b**) $\dot{\varepsilon }$ = 0.01 s^−1^; (**c**) $\dot{\varepsilon }$ = 0.1 s^−1^; (**d**) $\dot{\varepsilon }$ = 1 s^−1^.

Figure 4 displays the true stress–strain curves of the 0.6Zn and 1.1Zn alloys at 380~500 °C under different strain rates. As the strain increases under various deformation conditions, the stress first quickly rises, reaches its peak, then gradually falls and stabilizes. The variation in stress with strain reflects the variations microstructure. The dislocation slip, multiplication, and tangle result in work hardening and a constant increase in stress throughout the early stages of the deformation process. As deformation increases, the tangled dislocation further promotes the creation of dislocation cell walls and the production of sub-grains, which expand into recrystallized grains. After recrystallization, the dislocation density within the alloy rapidly decreases, the alloy softens, and the rate of increase in the flow stress begins to slow. The stress reaches its maximum when the rates of deformation introducing dislocations and dynamic recrystallization consuming dislocations are equal. The softening effect of dynamic recrystallization then intensifies and leads to a reduction in rheological stress. As deformation progresses, dislocation multiplication and dislocation tangle occur in the recrystallized grains, causing further work hardening. Following this, the effects of work hardening and dynamic recrystallization softening alternately increase, eventually reaching dynamic equilibrium and the rheological stress is essentially stable. As evidenced by the fact that flow stress reduces as the temperature and strain rate increase, both alloys are positive strain-rate-sensitive materials. Additionally, it has been discovered that, under most conditions, in comparison to the 0.6Zn alloy, the 1.1Zn alloy exhibits higher flow stress early in the deformation process, but lower flow stress late in the deformation process due to a faster rate of stress decrease with deformation. This distinction is unmistakably caused by the part that the LPSO phase plays in work hardening and dynamic recrystallization at various stages. In the pre-deformation stage, the hindering effect of the LPSO phases on the dislocation motion in the 1.1Zn alloy leads to more severe work-hardening, but due to the coordinated deformation effect of LPSO phases and the role of their interface as a favorable nucleation site for dynamic recrystallization, the softening rate in the later stage is much faster. The effect of temperature on the strengthening of the LPSO phases is not singular. At low temperatures, the LPSO phases coordinate the plastic deformation and release the stress concentration mainly through deformation, while at high temperatures, the dislocation plugging region near the interface of the LPSO phases serves as a nucleation location for dynamic recrystallization and promotes the occurrence of dynamic recrystallization, thus reducing flow stress.

**Figure 5:**
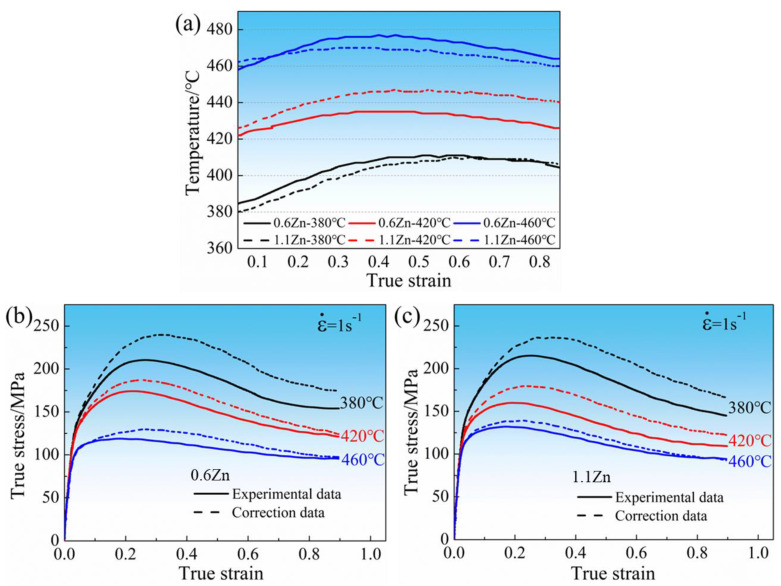
Temperature and stress correction for 0.6Zn and 1.1Zn alloys at a strain rate of $\dot{\varepsilon }$ = 1 s^−1^ (**a**) comparison of the real and preset temperatures; (**b**,**c**) comparison of measured stresses and the corrected stresses at the predetermined temperature for 0.6Zn and 1.1Zn alloys, respectively.

The real temperature of magnesium alloy during hot working is usually significantly higher than the set temperature at high strain rates, especially at lower temperature deformation, in which case it is particularly challenging for alloys to instantly consume energy via dynamic recovery and dynamic recrystallization [29]. It influences the stress and demands a flow stress rectification according to Equation (1) [30].

(1) $$\sigma_{0}=\frac{T_{1}}{T_{0}}(1+\frac{\Delta T}{T_{m}-T_{1}})\;\sigma_{1}$$

where *σ*_0_ and *σ*_1_ represent the stress at the preset temperature and the actual measured stress, respectively. *T*_1_, *T*_0_, *T_m_*, and Δ*T* are the real temperature, preset temperature, melting point temperature, and gap between *T*_0_ and *T*_1_, respectively. Figure 5a shows the comparison between the real and preset temperature of two alloys when compressed at a strain rate of $\dot{\varepsilon }$ = 1 s^−1^. Figure 5b,c, on the other hand, compares the actual measured stresses and the corrected stresses for compression at the predetermined temperature for 0.6Zn and 1.1Zn alloys, respectively. Clearly, the corrected stress is greater than the measured stress.

#### 3.2.2. Activation Energy

The Arrhenius equation [31], written as Equation (2), is frequently used to describe the relationship between the deformation temperature T, strain rate $\dot{\varepsilon }$, and flow stress σ of magnesium alloys during thermal deformation. Equation (2) is then transformed into Equation (3) by applying natural logarithms to both ends.

(2) $$\dot{\varepsilon }={A\left[\sinh\left(\alpha \sigma \right)\right]}^{n}exp(-\frac{Q}{RT})$$

(3) $$ln\dot{\varepsilon }=lnA+nln[\sinh\left(\alpha \sigma \right)]-\frac{Q}{RT}$$

where *Q* is the activation energy (kJ/mol), *R* is the gas constant (8.314 J/mol·K), *T* is the absolute temperature (K), σ is the stress value (MPa) at a given strain, and *A*, *n*, and *α* are the material constants.

Equation (4) at low stresses and Equation (5) at high stresses can also be used to demonstrate the connection between the rheological stress *σ*, strain rate $\dot{\varepsilon }$, and deformation temperature *T*, where *α* = *β*/*n*.

(4) $$ln\dot{\varepsilon }=lnA_{1}+n_{1}ln\sigma -\frac{Q}{RT}$$

(5) $$\;ln\dot{\varepsilon }=lnA_{2}+\beta \sigma -\frac{Q}{RT}$$

**Figure 6:**
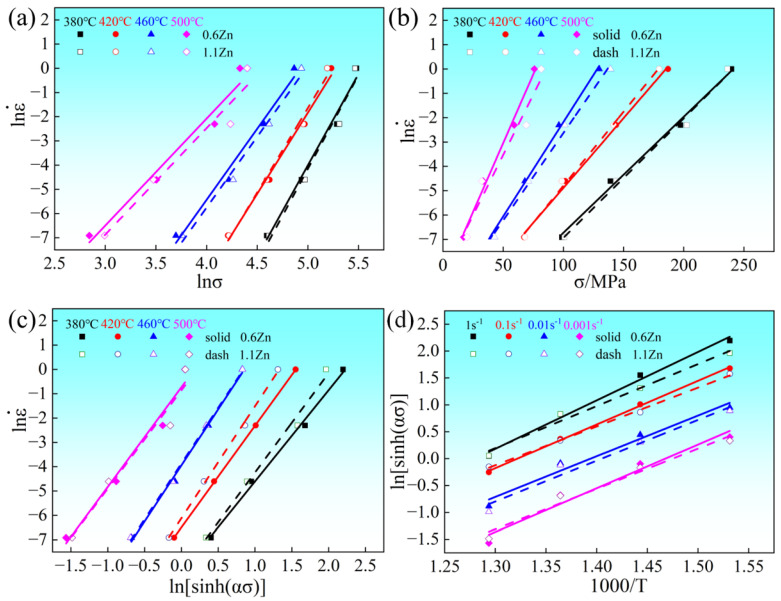
The plots constructed at maximum flow stress for the alloys (**a**) ln$\dot{\varepsilon }$-ln*σ*; (**b**) ln$\dot{\varepsilon }$-*σ*; (**c**) ln$\dot{\varepsilon }$-ln[sinh(*ασ*)]; (**d**) ln[sinh(*ασ*)]−1000/*T*.

The rheological activation energy *Q* under maximum stress can be calculated using Equation (2). Firstly, linear fitting curves of ln$\dot{\varepsilon }$-lnσ and ln$\dot{\varepsilon }$-σ can be obtained from the stress and strain rate values at different temperatures, and the slopes of the curves at different temperatures represent corresponding values of *n*_1_ and *β*, based on Equations (4) and (5), as illustrated in Figure 6a,b, respectively. Subsequently, the values of α are determined based on equation *α = β/n*_1_. Further, linear fitting curves of $ln\dot{\varepsilon \;}-$ln[sinh(ασ)] and $ln[\sinh\left(\alpha \sigma \right)]-1/T$ can be made, based on the values of the strain rate and flow stress, as well as the values of the flow stress and temperature, as shown in Figure 6c,d, and the slopes of the curves at different temperatures in Figure 6c and different strain rates in Figure 6d are symbolized by n’ and s, respectively. Q at maximum stress can then be determined as Equation (6):

(6) Q=R∂lnε˙∂ln⁡sinh⁡ασT{∂ln⁡sinh⁡ασ∂1T}ε˙=RNS

where N and S are average values of n’ corresponding to different temperatures in Figure 6c and s corresponding to different strain rates in Figure 6d, respectively. It should be pointed out that the linear correlation coefficients of the data are all higher than 98%. In Table 1, values of n_1_, α, β, N and S for 0.6Zn and 1.1Zn alloys measured are listed. The Q values for the 0.6Zn alloy and 1.1Zn alloy then measured are 293.45 KJ/mol and 279.48 kJ/mol, respectively. This finding is particularly intriguing because the LPSO phase is generally believed to contribute to the high Q value, attributed to the hindrance effect of the LPSO phase on the thermal movement of dislocations [32]. But in fact, in addition to the LPSO phase, RE solutes in the matrix can also contribute to the high Q value due to their segregation effect around moving dislocations [27]. Considering that the total RE content in the two alloys is essentially equal, whereas the LPSO phase is enriched with RE elements, the increase in the Q value due to RE solutes in the matrix is lower in the 1.1Zn alloy than in the 0.6Zn alloy. Therefore, it can be inferred that more Gd and Y atoms dissolved in the 0.6Zn alloy matrix contribute more to the Q value compared to the bulk LPSO phase in the 1.1Zn alloy, and result in a higher Q value for the 0.6Zn alloy.

### 3.3. Processing Maps

The thermal processing map, which combines a power dissipation diagram representing microstructure evolution during thermal compression and an instability diagram representing the unstable flow, is typically used to characterize the hot working property of metal materials. The total dissipated power (*P*) may be separated into two portions using a dynamic material model (DMM), as illustrated below [33]:

(7) $$P=\sigma \dot{\varepsilon }=G+J=\int_{0}^{\dot{\varepsilon }}\sigma d\dot{\varepsilon }+\int_{0}^{\sigma }\dot{\varepsilon }d\sigma$$

where *G* and *J* represent the power consumption related to the compression deformation and development of microstructures, respectively. The strain rate sensitivity exponent m represents the amount of energy dissipation caused by the evolution of the material’s internal microstructure during compression. It is a useful metric for characterizing metal plastic deformation and may be represented as [34]:

(8) $$m=\frac{dJ}{dG}=\frac{\partial \;ln\sigma }{\partial \;ln\dot{\varepsilon }}$$

Further, a power dissipation factor *η* is established to describe the proportion of power dissipated in microstructure evolution to the total power [35]:

(9) $$\eta =\frac{J}{J_{max}}=\frac{2m}{m+1}$$

**Figure 7:**
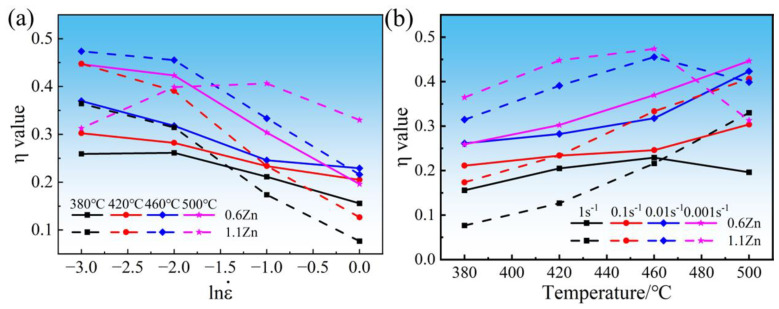
The variation curves of η values for 0.6Zn and 1.1Zn alloys with experimental strain rate (**a**) and temperature (**b**) during hot compression.

The fluctuation in η with the strain rate and temperature is then used to plot the power dissipation map. Figure 7 plots the variation curves of η values for 0.6Zn and 1.1Zn alloys with an experimental strain rate and temperature during hot compression. From Figure 7a, a similar pattern exists for the curves at 380 °C, 420 °C, and 460 °C. The main manifestation is that the 1.1Zn alloy has much higher *η* values than the 0.6Zn alloy at the 0.001 s^−1^ strain rate, but the difference between the two values decreases as the strain rate increases, and finally the η value of the 1.1Zn alloy falls below that of the 0.6Zn alloy. According to Figure 7b, at 380 °C, the η values of the 1.1Zn alloy are higher than those of 0.6Zn at 0.001 s^−1^ and 0.01 s^−1^, but lower than those of 0.6Zn at 0.1 s^−1^ and 1 s^−1^; at 420 °C, the 1.1Zn alloy has higher η values than 0.6Zn at 0.001 s^−1^ and 0.01 s^−1^, almost equal to 0.6Zn at 0.1 s^−1^, and lower than 0.6Zn at 1 s^−1^; whereas at 460 °C, the 1.1Zn alloy has higher η values than 0.6Zn at 0.001 s^−1^, 0.01 s^−1^, and 0.1 s^−1^, but lower than 0.6Zn at 1 s^−1^. These observations indicate that the promotion of dynamic recrystallization by the LPSO phases is related to the strain rate and temperature, and the LPSO phases promote dynamic recrystallization when the temperature is located at lower strain rates of 380~460 °C. However, as the strain rate increases, the promotion of LPSO phases in dynamic recrystallization weakens, and it is even inhibited at high strain rates. At 500 °C, a completely opposite pattern emerges. The η value of the 1.1Zn alloy is less than that of 0.6Zn at 0.001 s^−1^, but greater than that of 0.6Zn at 0.01 s^−1^, 0.1 s^−1^, and 1 s^−1^, and the gap grows with the increasing strain rate.

**Figure 8:**
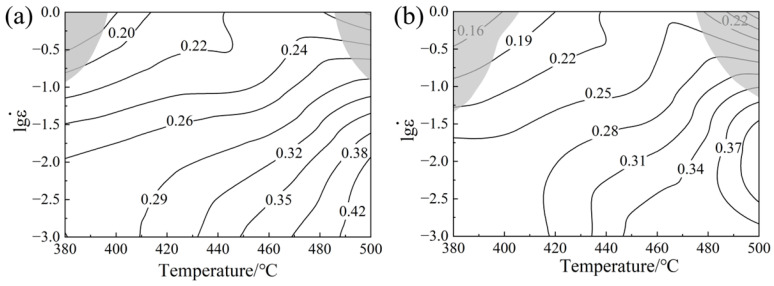
Processing maps of alloys (**a**) 0.6Zn; (**b**) 1.1Zn.

Furthermore, Ziegler’s thermal deformation extreme principle defines a continuous criterion taking the occurrence of flow instabilities into account [36], that is

(10) $$\xi =\frac{\partial ln(m/(m+1))}{\partial \;ln\dot{\varepsilon }}+m<0$$

where *ξ* denotes the instability coefficient, and the fluctuation in *ξ* with the strain rate and temperature is used to plot the instability map. The domain of instability is represented by *ξ* < 0, and the domain of safety is represented by *ξ* > 0. The thermal processing map of 0.6Zn and 1.1Zn alloys is then obtained by superimposing the instability map and the power dissipation map, as depicted in Figure 8. The values on the contour line indicate the power dissipation factor, and the shaded part represents the instability zone. It is clear that alloys exhibit instability zones around 380 °C and 500 °C at strain rates of 1 s^−1^, while the 1.1Zn alloy has a larger instability zone than the 0.6Zn alloy. In the safe domain, the power dissipation factor of both alloys increases with decreasing strain rates and increasing temperatures, meaning that the workability is improving. However, when compared to the 0.6Zn alloy, the power dissipation factor for the 1.1Zn alloy is lower at low strain rates and elevated temperatures. The maximum power dissipation factor of the 0.6Zn alloy is located at 500 °C, between 0.01 s^−1^ and 0.001 s^−1^, whereas the maximum power dissipation factor of the 1.1Zn alloy is located at 500 °C, 0.01 s^−1^ and its vicinity, with values above 0.4, corresponding to the optimal thermal processing windows.

### 3.4. Microstructure Analysis of Characteristic Regions

#### 3.4.1. Microstructures with Variation in η Value

**Figure 9:**
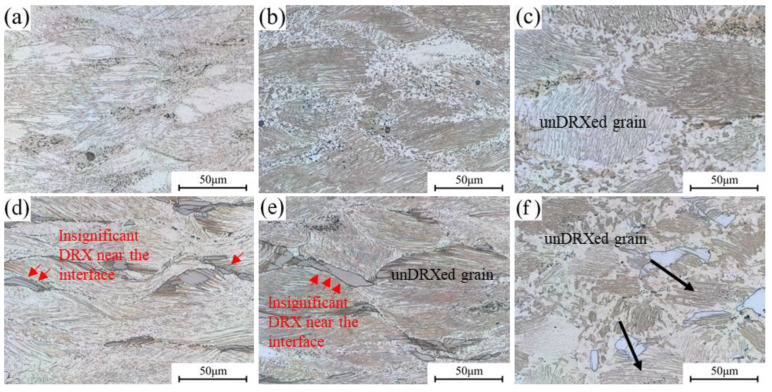
Microstructures of the samples under different strain rates at 420 °C (**a**) 1 s^−1^, 0.6Zn; (**b**) 0.1 s^−1^, 0.6Zn; (**c**) 0.001 s^−1^, 0.6Zn; (**d**) 1 s^−1^, 1.1Zn; (**e**) 0.1 s^−1^, 1.1Zn; (**f**) 0.001 s^−1^, 1.1Zn.

The microstructures of 0.6Zn and 1.1Zn alloys at 420 °C at various strain rates are illustrated in Figure 9. When the strain rate is 1 s^−1^ for the 0.6Zn alloy, as shown in Figure 9a, the grains experience obvious bending deformation based on the bending of lamellar LPSO phases that forms during the hot compression process [27]; many deformation bands appear, and tiny dynamic recrystallized grains show up around the grain boundaries and deformation bands. As the strain rates increase, the dynamic recrystallization increases, and the bending of deformed grains is weakened. The 1.1Zn alloy exhibits a similar pattern to the 0.6Zn alloy in that as the strain rates decrease, the dynamic recrystallization increases. LPSO phases undergo significant plastic deformation such as kinking and bending at low strain rates, which reduces the dislocations accumulation near the LPSO phases. This is supported by the red arrows in Figure 9d,e, which show that the degree of recrystallization at the interfaces between the α-Mg matrix and the bulk LPSO phase is lower than that at other grain boundaries. Therefore, the 1.1Zn alloy’s overall dynamic recrystallization ratio is less than 0.6Zn at low strain rates. However, when the strain rate drops to 0.001 s^−1^, a greater degree of dynamic recrystallization occurs in the 1.1Zn alloy, with a smaller proportion and size of undynamically recrystallized grains, as indicated by the black arrows. It is primarily due to the fact that the deformation primarily occurs in Mg grains with a lower critical shear stress for the dislocation slip and thus the LPSO phase interfaces act as the nucleation core for dynamic recrystallization.

**Figure 10:**
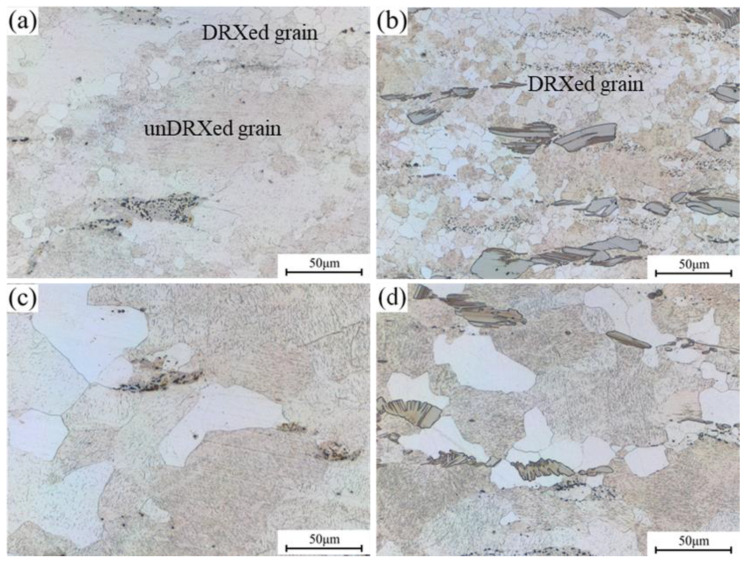
Microstructures of the samples with experimental strain rates varying at 500 °C (**a**) 0.1 s^−1^, 0.6Zn; (**b**) 0.1 s^−1^, 1.1Zn; (**c**) 0.001 s^−1^, 0.6Zn; (**d**) 0.001 s^−1^, 1.1Zn.

The microstructure of 0.6Zn and 1.1Zn alloys with experimental strain rates varying at 500 °C is depicted in Figure 10. The 0.6Zn alloy exhibits apparent dynamic recrystallization along the grain boundaries when the strain rate is 0.1 s^−1^, but coarse grains without dynamic recrystallization still make up a significant fraction. It appears that a higher temperature aids LPSO phases in acting as a promoter of dynamic recrystallization because a higher degree of dynamic recrystallization took place in the 1.1Zn alloy and the recrystallized grains were finer and more uniform, which is substantially different from that at the same strain rate at 420 °C as shown in Figure 9e. Both alloys have quite coarse grain sizes when the strain rate is 0.001 s^−1^, which should be the result of a long-term high temperature that promotes the growth of dynamic recrystallized grains. But as a result of the combined actions of LPSO phases as the nucleation point to refine recrystallized grains and suppress grain expansion by pinning grain boundaries, the grain sizes of the 1.1Zn alloy are comparatively smaller in contrast. Further, it could be inferred that the main processes including dynamic recrystallization and grain growth were implemented earlier in thermal compression process of 0.001 s^−1^ for the 1.1Zn alloy, and the energy required for microstructure evolution in the later stage is substantially smaller; thus, the overall power dissipation factor is less than 0.6Zn. This coincides with the optimum thermal processing parameters in processing maps (Figure 8b) for the 1.1Zn alloy.

#### 3.4.2. Microstructure Analysis in Instability Regions

**Figure 11:**
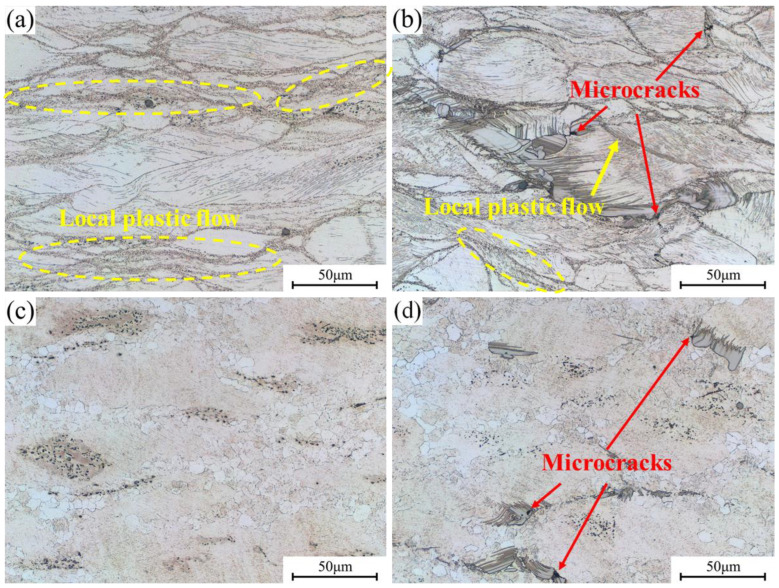
Microstructures of instability regions in processing maps for 0.6Zn and 1.1Zn alloys (**a**) 380 °C/1 s^−1^, 0.6Zn; (**b**) 380 °C/1 s^−1^, 1.1Zn; (**c**) 500 °C/1 s^−1^, 0.6Zn; (**d**) 500 °C/1 s^−1^, 1.1Zn.

Figure 11 depicts the microstructure of 0.6Zn and 1.1Zn alloys in two instability regions at 380 °C/1 s^−1^ and 500 °C/1 s^−1^. When under 380 °C/1 s^−1^, it can be seen that many shear bands, which exhibit strongly non-uniform deformation and contain a large number of extremely fine grains, pass through multiple grains in both alloys. It indicates that plastic deformation has been localized, and allows for stress concentration and the formation of defects like microcracks and micropores. As seen in Figure 11b, α-Mg and bulk LPSO phases suffered severe deformation, and the non-uniform deformation of the 1.1Zn alloy eventually resulted in microcracks at the interfaces of the α-Mg and bulk LPSO phases due to stress concentration. While under 500 °C/1 s^−1^, the 0.6Zn alloy contains clear mixed grains with dynamically recrystallized grains mainly at the grain boundaries, and the dynamic recrystallized grain size has grown to about 6μm. This is primarily because, in the event of a very brief deformation time, a significant amount of heat is produced inside the sample of the Mg-Gd-Y-Zn alloy with low thermal conductivity, which is no time to be released to the surface of the specimen, thus encouraging dynamic recrystallization and grain growth. But for the 1.1Zn alloy, the internal deformation heat that cannot be released in time not only causes grain growth but also increases the heating in the LPSO phases, thus resulting in local liquefaction and the formation of pores.

## 4. Conclusions

In this work, two alloys of 0.6Zn without the LPSO phase and 1.1Zn alloy with the bulk LPSO phase were prepared, and the thermal compression behaviors and microstructures of the two alloys were studied to clarify the effects of LPSO phases on the thermal activation energy, thermal workability, and dynamic recrystallization of the alloys during thermal compression. The findings shed some new light on the understanding of the role of LPSO phases in the hot deformation process and offer recommendations for the composition and process design of Mg-RE-Zn alloys containing LPSO phases. However, there is still some further in-depth and detailed analysis required by more advanced analytical methods to complete the underlying mechanism of the influence of the bulk LPSO phase on the rheological stress and microstructure evolution. Specifically, some important findings are as follows:

1. Increasing the strain rate or decreasing the compression temperature can improve the flow stress for the two alloys, but the 1.1Zn alloy exhibits higher flow stress early in the deformation process, but lower flow stress late in the deformation process due to a faster rate of stress decrease with deformation.

2. The 1.1Zn alloy with bulk LPSO phases exhibits a higher Q value than the 0.6Zn alloy without the LPSO phase (279.48 vs. 293.45 kJ/mol), suggesting that more Gd and Y atoms dissolved in the 0.6Zn alloy matrix contribute more to the rheological activation energy compared to the bulk LPSO phase in the 1.1Zn alloy.

3. When compressed in 380~460 °C, the dynamic recrystallization is promoted by bulk LPSO phases at lower strain rates but inhibited at high strain rates. While at 500 °C, the LPSO phase promotes dynamic recrystallization at all experimental strain rates.

4. The optimal processing windows of the 0.6Zn and 1.1Zn alloys are in the regions of 500 °C/0.001~0.01 s^−1^ and 500 °C/0.01 s^−1^, respectively. The instability zones for the 1.1Zn alloy are larger than that for the 0.6Zn alloy, which corresponds to the microcracks generated at the interfaces between the α-Mg and bulk LPSO phases.

## Figures and Tables

**Table 1:** The average measured values of *n*_1_, *α*, *β*, *N* and *S* for the alloys.

Alloy	*n*_1_	*α*	*β*	*N*	*S*
0.6Zn	6.133	0.012	0.074	4.158	8.208
1.1Zn	6.238	0.011	0.070	4.328	7.530

## Data Availability

Not applicable.
